# The Sustainable Development Goals: Contextualizing Africa's Economic and Health Landscape

**DOI:** 10.1002/gch2.201800014

**Published:** 2018-06-21

**Authors:** Marlon E. Cerf

**Affiliations:** ^1^ Biomedical Research and Innovation Platform South African Medical Research Council PO Box 19070 Tygerberg Cape Town 7505 South Africa

**Keywords:** gross domestic product, healthcare human capital, health landscape, inequality, poverty, responsible trade, subsidizing health

## Abstract

The sustainable development goals (SDGs) encompass 17 goals with targets and indicators, collectively striving to improve national, regional, continental, and global development. SDG 8 strives for improved and sustainable economic growth. Africa's population is estimated to increase markedly and rapidly over the next few decades. The African demographic dividend presents opportunities to be harnessed, but several socioeconomic challenges exist that may constrain progress for achieving the SDGs. Poverty and inequality are pervasive in Africa and constrain economic and health gains. SDG 3 aims for good health and well‐being for all ages and has 13 targets linked to 26 indicators. Collectively, SDG 3 targets aim to improve health outcomes by reducing mortality, ending epidemics, and preventing diseases to ensure affordable and quality healthcare access for all. The dynamic African health landscape and scarcity of healthcare human capital also present challenges for advancing SDG 3. The implementation of the SDGs presents major and complex challenges but ultimately yields rewards. Advancement across all SDG 3 targets is necessary for the benefit of healthier global citizens.

## 1 Introduction

The sustainable development goals (SDGs) are integrated, indivisible, and balance the three pillars of sustainable development, viz., the economic, social, and environmental while also adopting the principles of people, planet, prosperity, peace, and partnership.^1^ Public accountability and participation in priority setting^2^ are required for the successful implementation of the SDGs. The World Health Organization (WHO) has a unique mandate and strongly advocates for all people to have access to healthcare.^3^ SDG 3, that aims to ensure healthy lives and promote well‐being for all at all ages, comprises 13 targets and 26 indicators, the most indicators of all the 17 SDGs.^4^ Target 3.8 of SDG 3 on universal health coverage (UHC) emphasizes that all people and communities should have access to quality health services without financial hardship.^1^ Health services should ultimately target individuals by encompassing curative care and health promotion.^5^ Health investments advance life expectancy, healthier workers, and economic productivity.^6^ UHC is a major global political and ethical health goal embracing the key ethical concepts of fairness, equity, and benefit, concomitant with solidarity justifying the mutual determination for achieving UHC^3^ nationally, regionally, continentally, and globally. However, the progress in achieving the targets of SDG 3 in Africa presents major challenges, given the socioeconomic constraints and the dynamic health landscape.

Although there are opportunities to be harnessed in Africa, there are important socioeconomic development priorities and challenges that need to be addressed. **Table** **1** expands on some of Africa's socioeconomic priorities and challenges discussed in an African Development Bank report on inclusive growth.^7^ Africa's socioeconomic priorities are already aligned with the SDGs to foster continental progress and avoid duplication. Africa's top social and economic priority and challenge is providing adequate healthcare (SDG 3) and sustainable economic growth (SDG 8), respectively (Table 1). Therefore, defining these i) socioeconomic and ii) health challenges and priorities in Africa is critical for informing the advancement on SDG 3. Perspectives on Africa's economy (SDG 8) and health (SDG 3) will be presented, as they are top priorities for continental advancement (Table 1). Some of Africa's other priorities will be briefly discussed in the context of health and economy where relevant, such as poverty and inequality.

**Table 1:** Africa's socioeconomic priorities and challenges aligned with the sustainable development goals

Social priorities and challenges	SDG	Economic priorities and challenges	SDG
Health	3	Economic growth, jobs, and structural transformation	8
Education	4	Agriculture, food, and nutrition security	2, 1
Water, sanitation, and access to basic services	6, 7	Energy	7
Demography and population growth	9, 11	Sustainable consumption and production	12
Urbanization and sustainable human settlements	11	Infrastructure development	9
Poverty, inequality, and social exclusion	1, 5, 10		
Youth unemployment	8		
Gender and women's empowerment	5		

## 2 African Socioeconomic Challenges and Priorities

### 2.1 Economic Growth

Africa's population is estimated to increase from 1 billion to 1.6 billion by 2030, doubling by 2050.^7^ Africa's gross domestic product (GDP) is estimated to grow 11–15% from 2011 to 2030 and with GDP and GDP per capita predicted to progressively rise from 2010 to 2060.^7^ By then, most African countries are anticipated to transition to upper middle income status with extreme poverty eliminated.^7^ However, there are considerations on Africa's estimated economic growth and the elimination of poverty. Previously, Africa's development was driven by high prices for export commodities, the discovery and exploitation of natural resources, stability, more robust governance in select countries, and the maturing demographic dividends.^8^ However, with the collapse of global oil and other mineral prices, growth in sub‐Saharan Africa decelerated to 1.5% in 2016, the lowest level since the 1980s, with the region's real GDP per capita contracting by 1.1%; South Africa and oil‐exporting countries mostly accounting for the slowdown.^9^ Therefore, the projected GDP growth in Africa may require adjustment as the commodity boom recedes unless there is value added to the commodities.

African countries have not embraced manufacturing opportunities to foster growth and development and remain marginalized in global manufacturing trade^10^ as the commodity export boom of the mid‐2000s did not prompt the diversification of their economies from commodity dependence to value adding manufacturing products^11^ and services. African countries' economies should transition from activities that provide diminishing returns over time, e.g., agriculture, mining, logging, and fishing toward activities that provide increasing returns over time, e.g., manufacturing and services to reach their full potential.^12^ Failure to progress along the more sustainable manufacturing and services economic growth trajectory will shackle African countries' economies and they will continue to lag behind other developing and more developed countries, thereby perpetuating and further widening the gap of poverty and inequality.

### 2.2 Poverty, Inequality, and the Right to Health

Poverty (targeted by SDG 1) in West, East, Southern, and Central Africa is extensive with large inter‐ and intracountry inequalities (targeted by SDG 10).^13^ Sub‐Saharan Africa remains the world's poorest region and faces extensive challenges in meeting the SDG targets^14^ with a poverty rate of 48.5% and >50% of the population of most countries living below the poverty line for ≥10 years.^15^ In Africa, there has been slow progress to reduce poverty and inequality due to limited decent employment opportunities (targeted by SDG 8) and weak social insurance mechanisms.^13^ Poverty and inequality are intertwined and should be addressed in tandem.^13^ Poverty disempowers individuals, cripples nations, and widens inequality. Most people in the world's top decile are from prosperous nations, not African countries.^16^ The quality of life and the rising expectations of the 20% of the world's wealthy, who consume 80% of our global energy and resources, seemingly ignore that our current global health, economic, social, and environmental dilemmas are attributed to entitlement and wasteful consumption patterns,^17^ in conflict with SDG 12 that purports responsible consumption. The eradication of poverty requires a shift from global economic growth to better and fairer distribution of the benefits of global production and consumption,^18^ which requires a reduction in the overconsumption of the world's wealthy.^19^ The SDGs recognize poverty as a complex, structural problem but explicitly avoid affirming that reduced consumption by the world's wealthy and regulating corporate extraction is necessary.^20^ The eradication of poverty through fair distribution, reduced consumption, and corporate regulation presents a mammoth task that will take generations to implement, if at all possible, and is a contentious issue to address. In essence, the SDGs aim to reduce inequality by improving the situation of the poor, while preserving the wealth and power of the global rich 1%.^20^ Hence, the cycle of poverty cannot be solved without challenging the pathologies of accumulation and wasteful, irresponsible consumption as eradicating poverty requires deep, structural transformation.^20^ Structural reforms should also address distortions in the labor market, which is marked by gender and other discriminatory patterns such as low pay relative to the skills offered, and also drive the development of more efficient delivery systems, coupled with more accountability.^21^ The extreme complexity of SDG implementation requires constant study, debate, and working partnerships to realize these ambitious global goals.

Economic growth, poverty, inequality, and responsible consumption are addressed by the SDGs. However, human rights which are associated with these SDGs and with SDG 3 that promotes good health for all, are silent in the SDGs. Inequities in health and access to healthcare persist, undermining economic growth^21^ in many African countries. The right to health refers to the entitlement of all humans to organized efforts by society that promote and improve health and the corresponding obligations of governments and the international community, as enshrined in international human rights law.^22^ The representations of the right to health in the SDGs are considered insufficient and superficial as there are no direct commitments or right to health discourse.^23^ The indicators required for UHC monitoring in the SDGs that incorporate the legal principles to the right to health include progressive realization of the right to health and fulfillment of the minimum core obligations, cost‐effectiveness, nondiscrimination, shared responsibility, participatory decision making, and attention to vulnerable and marginalized groups.^24^ However, the right to health also requires the rights to water and sanitation, food, housing, education, and collectively, to development.^23^ Therefore, without basic human needs being met, the right to health cannot be achieved. As human rights are vaguely embedded and not lucidly defined in the SDGs, it is imperative to bring human rights, and the right to health for SDG 3, to the fore during the SDG era so that cognizance and awareness prevail and the most vulnerable people are not forgotten.

### 2.3 Responsible Trade

Responsible trade refers to fair and equitable trade between countries during the production of goods and services. Given the potential overestimation of future economic growth and the persevering poverty coupled to inequality, African policy makers should explore protectionism policies^25^ to guard Africa's resources and advance its growth. Trade agreements need to favor the African country, as the supplying nation, and not replicate recolonization and the plundering of resources by “invading” nations. Of course, trade agreements and the global flow of goods and services represent free trade and enable growth. Maintaining diplomatic relations is obligatory, but fair trade should be fair: African countries are supplying raw materials, the value addition is mostly done by a non‐African country, and the African country receives a pittance relative to its “aiding” counterpart who profits from an African resource. Responsible trade is where the entering country aids and skills the African country to add value to its own goods and services. African countries then develop faster by becoming better equipped and more economically independent, thereby enabling growth for sustaining their domestic economies. This helps to reduce poverty and inequality through decent job creation that feeds into a virtuous cycle that accelerates national, regional, and continental advancement and ultimately enables African countries to transition to manufacturing and services' value added economies to foster growth.

### 2.4 Africa's Demographic Dividend

One route to faster economic growth, and to reduce poverty and inequality, is to carefully harness Africa's demographic dividend to drive prosperity. Continual rapid growth in the economically active population (men and women aged 15–64), at ≈3.5% per annum, will enlarge Africa's working age population of ≈1.87 billion, with ≈74% of Africans of working age.^7^ Economic and population growth will be associated with rapid migration and urbanization.^7^ The transitioning of working age individuals from rural to urban areas will accelerate economic growth.^7^ However, with increasing urbanization, infrastructural demands and pollution increase, and with higher density, the incidences of communicable diseases and outbreaks have higher probabilities. Natural events and disasters have devastating health and economic consequences. For example, a severe drought can contribute to increasing infections (due to water scarcity and impurity) and exacerbate poverty (due to higher food inflation), whereas cyclones and flash flooding prompt the prioritization and reallocation of public funds for aiding displaced individuals and for restoring infrastructure sometimes at the expense of healthcare and welfare. Noncommunicable diseases (NCDs) will continue to rise with increasing urbanization, given the dietary and lifestyle changes after transitioning from more rural environments to urban areas, a consequence of economic growth.

Africa's population was estimated at 1.0 billion in 2010, peaking at 2.7 billion in 2060.^7^ This delayed demographic transformation will translate in Africa benefiting from a demographic dividend, i.e., the increase in the ratio between the working age population and the nonworking age population.^7^ About 50% of Africa's population is aged ≤17, with the active population aged 15–64 predicted to triple between 2005 and 2060.^7^ This demographic dividend will likely increase the labor force, reduce dependency ratios, augment national savings, and accelerate urbanization, translating into higher productivity and faster economic growth.^7^ However, this potential virtuous cycle is dependent on other factors, viz., a well‐educated labor force, efficiently mobilized national savings, and the adoption of suitable economic policies and good governance.^26^ In countries with high fertility rates, a lower probability of child mortality can positively influence household decisions on family planning and thereby contribute to a faster demographic transition and the associated economic benefits of the demographic dividend.^21^ The demographic dividend is to be harnessed effectively to ensure the economic progression of African nations, e.g., from low‐income to middle‐income countries, through advancement toward manufacturing‐ and services‐based economies. However, development across Africa is not uniform and some African countries will inevitably transition more rapidly. Socioeconomic disparities dictate the rate of development across African countries. The key levers for achieving this positive transition is in a well‐educated workforce with the adequate and relevant skills to contribute to the GDP; and to have a population that adopts a healthy lifestyle to prevent or delay the onset of diseases and reduce dependencies on health services.

## 3 African Health Challenges and Priorities

### 3.1 African Health Landscape

The dynamic African health landscape is shaped by global health trends and continental socioeconomic constraints. Some important considerations on the global health landscape are the emergence of NCDs, as a group (notably cardiovascular disease, cancer, diabetes, and respiratory diseases), as the main burden of disease globally; the increasing significance of mental health (often clustered with NCDs); human immunodeficiency virus/acquired immunodeficiency syndrome (HIV/AIDS) evolving into a chronic disease; comorbidities becoming more prevalent; drug resistance challenges in tuberculosis (TB); the rising prevalence of antimicrobial resistance; healthcare worker, funding, and infrastructural resource constraints; and the adoption of precision medicine for better health treatment through enhanced and specific treatment of diseases per region across ethnicities.

The impact of good investments and effective interventions in Africa are becoming increasingly evident. However, the high burden of diseases concomitant with the scarce healthcare resources remains overwhelming challenges. HIV/AIDS, TB, and malaria pose the greatest challenges. There is the emergence of the increasing burden of both morbidity and mortality from NCDs. Chronic diseases are more prevalent, linked to demographic, behavioral, and social factors. Socioeconomic factors influence disease burdens and health service delivery. There are changes in lifestyles and increasing urbanization. Hypertension, stroke, diabetes, chronic respiratory diseases, and substance abuse are becoming more prevalent. There is worsening protein, caloric, and micronutrient malnutrition in many countries that contributes to increasing morbidity and mortality. Dietary changes and inactivity are driving the emergence of chronic diseases of lifestyle and their precursors such as obesity, insulin resistance, and metabolic syndrome.

A critical success factor for health system delivery is learning and providing support across Africa with early warning systems to prevent and respond to outbreaks. Health promotion and disease prevention is cheaper than treatment; it cost an estimated $4.7 billion to treat Ebola patients. The harmonization of health research priorities must be integrated into national research systems that can reach into a continent and therefore contribute to global health and disease estimates that are more timely and accurate. For effective health coverage in Africa, basic needs need to be met. These include good nutrition (SDG 2), family planning (empowering women; SDG 5), curing epidemics (SDG 3), providing decent jobs (including the informal sector; SDG 8), and alleviating poverty (SDG 1). Building adequate infrastructure for people (SDG 9) and maintaining an environment (SDGs 9, 11, 12) suitable for economic growth (SDG 8) and healthy lifestyles (SDG 3) are critical for ensuring that the health and well‐being of individuals within nations improve (SDG 3).

### 3.2 African Health Gains

Maternal mortality rates in Africa (excluding North Africa) declined 35% from 2000 to 2015, with Cape Verde, Tunisia, Libya, Egypt, and Mauritius achieving the SDG target of <70 deaths per 100 000 live births.^13^ From 2000 to 2015, there were also reductions in under‐5 (46%) and neonatal (30%) deaths.^13^ These positive trends are partly attributed to improved access to skilled birth attendants and family planning.^13^ HIV incidence in Africa declined ≈62% from 2000 to 2015.^13^

Through a holistic and integrative approach, Rwanda has achieved some remarkable health successes. Rwanda is an early adopter of technological and clinical innovations with its central government and the Ministry of Health credited for their health gains.^27^ The Rwandan government incorporates social support such funding travel and food in its national treatment programs for AIDS and tuberculosis.^27^ Further, the Rwandan Ministry of Health, in collaboration with 23 United States institutions, launched a unique and comprehensive program to enhance education and skills of their health workforce by building capacity of medical, nursing, and midwifery faculty.^28^ From inception, Rwanda's AIDS program integrated prevention and control, simultaneously addressed tuberculosis and malnutrition, and strengthened the healthcare system.^27^ Scale‐up of AIDS services began in cities and towns and later expanded into the more populated rural areas.^27^ In 2012, 108 113 Rwandans with advanced HIV received antiretroviral therapy, making Rwanda and the more affluent Botswana the only countries in sub‐Saharan Africa to achieve universal access to antiretroviral therapy.^29^

Like Rwanda, Ethiopia has also made important health gains through a partnership approach to efficiently deliver health services. Many development partners support Ethiopia to strengthen its health system and upgrade lifesaving health services.^30^ Collectively, donors contributed ≈$4.5 billion to Ethiopia's health sector development program, along with ≈$1.5 billion from the Ethiopian government.^30^ There were intensifying efforts to reach pregnant women and mothers to address Ethiopia's high maternal and neonatal mortality rates with >35 000 health workers deployed to rural areas.^30^ Deaths of children <5 years dropped from 123 per 1000 live births in 2005 to 88 in 2011.^30^ Further, Ethiopia had the greatest percentage decline in the maternal mortality ratio.^13^ These health gains were attributed to task sharing for maternal newborn child and adolescent health and family planning; increased political will through strong collaboration and partnerships; improved skilled birth attendance by expanding and strengthening the community health program; capacity building of midwives with improved midwifery education and implementing a midwifery workforce database; and reformed laws on abortion with wider access to safe abortion services.^13^

### 3.3 African Health Lag

Despite health gains in some African countries, major challenges persist such as disease outbreaks, poverty, inequality, and war that constrain health service delivery, thereby compromising healthcare for affected citizens. Twenty African countries reported a maternal mortality ratio of >500 deaths per 100 000 live births in 2015 with the highest maternal mortality in Sierra Leone at 1360 deaths per 100 000 live births.^13^ At the Medécins Sans Frontières (MSF or Doctors Without Borders) AIDS center in the Democratic Republic of Congo (DRC), the treatment costs contributed to late‐stage AIDS patients becoming progressively morbid yet still delayed seeking care.^31^ In the Central African Republic (CAR), people were deterred by healthcare costs, viz., $2.7 for HIV testing and $9 for a monthly supply of antiretroviral drugs.^31^ The unfortunate consequences are that many Central Africans remain untested for HIV, are only treated once, or give up on care.^31^ Healthcare has to be affordable or subsidized or it will severely constrain universal access as the most vulnerable people will experience financial hardship, contradictory to what UHC sets out to achieve.

Political stability is required^10^ for the advancement of the SDGs in Africa. Unfortunately, poverty, inequality, and war perpetually constrain health service delivery and stagnate health gains. In 2013, the CAR (conflict), South Sudan (conflict), and West African countries (Ebola outbreak) were categorized as humanitarian emergencies by the WHO.^32^, ^33^ In 2014, in the CAR, armed conflict disrupted the lives of ≈2.5 million people, uprooted ≈500 000, injured ≈7000, and destroyed health infrastructure, thereby delaying immunization and other preventive health programs, also greatly increasing malnutrition.^33^ In some regions in the CAR, >75% of the health facilities could not offer basic services; one‐third of the district hospitals were unable to provide emergency services; two‐thirds of immunization services were nonfunctional; and only a quarter of the ambulances were in service.^33^ This severe infrastructural and resource impairment in the CAR compromised the health service delivery and will have long‐lasting consequences on the population's health outcomes.

Since 2008, renewed fighting in the north‐east of the DRC displaced ≈250 000 Congolese exacerbating already tough situations for >1 million people living without clean water, sufficient food, or access to healthcare.^34^ Without meeting basic human needs, access to healthcare becomes extremely hard to attain. The DRC's health system suffered from a lack of investment, and was further degraded by deliberate attacks from factions^34^ as in the CAR.^33^ In the DRC, a cholera epidemic affected 10 332 people with 201 deaths.^34^ Other waterborne diseases, such as *Shigella*/dysentery are also major health burdens; and some Congolese children are weak and malnourished, rendering them susceptible to malaria, which is a major cause of pediatric death.^34^

### 3.4 Scarce African Healthcare Human Capital

There is a global shortage of health workers and a lack of skilled labor constrains job creation in the health sector.^21^ Targeted investment in health systems, including the health workforce, promotes economic growth along other pathways such as economic output, social protection and cohesion, innovation, and health security.^21^ Even as a leading economy in (sub‐Saharan) Africa, South Africa has healthcare human capital constraints. The health sector contributes to the economy by job creation, investing in infrastructure, and purchasing the required healthcare supplies.^6^ In South Africa, recent estimates of the number of physicians per 1000 population was 0.77 and 0.2 for nurses and midwives,^35^ but these ratios conceal the maldistribution within the private versus public sectors and urban versus rural areas.^36^ It is imperative that adequate and relevant resources are allocated according to demand, i.e., the disease burden per region; and are mobile, i.e., physicians are responsible for health services in geographical areas and not assigned to specific hospitals, especially in rural areas with large distances between hospitals. This benefits patients as they are treated appropriately and makes better use of the physician resources, given that they are scarce and provide an essential service.

The investment in decent health sector jobs enhances social protection systems, e.g., in morbidity, incapacity, unemployment, and old age; and offers financial protection against the lost income, out‐of‐pocket payments, and unplanned disastrous health expenses,^6^ in line with SDG 3 that strives for no financial hardship incurred through health expenses. In 2008, 33 534 medical practitioners (physicians) were registered with the Health Professions Council of South Africa.^36^ South Africa recruits from non‐Southern African Development Community (SADC) African countries: India, North America, and Europe; apart from bilateral agreements with Tunisia, Iran, and Cuba that allow these foreign physicians to work in South Africa.^37^ The oldest, most successful, and established bilateral agreement is the South African–Cuban program, embedded with an intentional oversupply of physicians.^37^ About 500 Cuban physicians work in South Africa at any given time, and their remittances are a source of foreign currency and taxable public revenue for Cuba.^38^

Unfortunately, many South African physicians emigrate, resulting in a loss of human capital. Hence, in these instances, South Africa is training physicians for export which it can ill afford. There are also new medical schools emerging at existing universities. The establishment of a Health Faculty that includes undergraduate training programs for medicine elevates a university's national status and brings in a new and major revenue stream. There are several under‐resourced universities in South Africa, an unfortunate legacy of apartheid. These universities need to expand or introduce health professional training programs to further boost local development. The bilateral physician exchange programs are beneficial for international relations and deployment into rural areas. However, more focus is required on training, upskilling, and retaining physicians and improving their working conditions. With triaging of duties, nonphysicians can attend to more routine cases and refer the more serious cases to the physicians. Degrees for these specialized medical assistants (nonphysicians), positioned between physicians and nurses, should be introduced as a new career path. This should be geared to train professionals to conduct basic diagnosis, prior to referral to the responsible physician. This will improve workflows in hospitals and clinics, thereby improving health service delivery and patient outcomes. A three‐year clinical officer training program implemented in Uganda, Kenya, Tanzania, and South Sudan covers public health, nursing, and surgical procedures, anatomy, pathology, pharmacology, orthopedics, psychology, and psychiatry with graduates employed in rural and urban areas.^39^ The program facilitated the delivery critical health services during and post civil war in Sudan and enhances national coverage through the deployment of the clinical officers throughout South Sudan, without urban bias. Such programs should be coordinated and rolled out throughout Africa to meet health demands and allow for specialist training to address the high priority disease burdens.

As the largest group of healthcare workers, nurses and midwives are the pillar of healthcare delivery in Africa.^40^ An estimated 620 000 nurses and midwives are needed in sub‐Saharan Africa, a region with 11% of the global population, 24% of the overall disease burden, 67% of global HIV, 3% of all healthcare workers, and <1% of global health expenditures,^41^ reflecting a clear imbalance of healthcare human capital resources, given the disease burden. Many African countries such as Kenya, Malawi, Zambia, and Zimbabwe mostly employ technicians instead of registered nurses.^42^ Shifting some tasks from people requiring longer and more intensive training, to those requiring shorter and less intensive training, enables more service availability within a shorter time.^43^, ^44^ The shortage of physicians in Africa, especially in rural areas, has therefore prompted task shifting toward nurses and midwives.^45^ However, these nurses and midwives are not properly trained and equipped for these additional tasks usually assigned to physicians.^45^ Nurses and midwives present a logical and viable source for enhancing health service delivery in Africa, thereby improving health outcomes for the continent's diverse population. There is a clear need to invest and train nurses and midwives for the correct skill mix to meet the domestic health demands in individual African countries that are aligned and reinforced regionally and continentally.

### 3.5 Subsidizing Healthcare and Health Research

Countries could afford universal access to an array of public health services provided mostly through policy, population‐wide, and periodic schedulable and outreach delivery platforms.^46^ An effective policy intervention to curtail the increasing incidence of NCDs globally and to decrease future expenditure on disease management^47^ is the implementation of taxes on goods that have adverse health implications such as tobacco, alcohol, sugar,^48^ fats, and processed food. From 1 April, 2018, the South African Revenue Service will tax manufacturers with a sugary beverages levy (at 2.1 cents (ZAR) per gram of the sugar content > 4 grams per 100 mL) to finance the prevention and control of NCDs and obesity.^49^

There should also be some incentives for adopting healthy nutrition and being physically active which translates into health promotion and disease prevention. For example, monetary rebates for healthy eating and reward for meeting activity targets are already implemented by medical aids (private medical insurance). This lends itself to strengthening public–private partnerships. Taxes derived from alcohol, tobacco, sugar, fat, and processed food can partially (albeit modestly) subsidize health services, fund health research, and sponsor health promotion initiatives, which contribute to the economy and help to reduce morbidity and mortality, e.g., from NCDs. Taxes from sugar, fat, and processed food can fund some NCD research, leading to policies that result in practices, or health innovations, to reduce the burden of the associated NCDs, e.g., diabetes, cardiovascular disease, cancer, and obesity—often a precursor to several NCDs. Thus, a decrease in morbidity and mortality translates in a reduced economic burden (i.e., absenteeism and lost productivity decreases, thereby freeing up health services as citizens are healthier). The reduction in a cluster of NCDs will alert industry and thereby inform industry on how to improve formulae that include healthy ingredients to benefit i) consumer health outcomes and ii) profitability as less taxes are due or tax rebates will be realized. This illustrates a virtuous public–private sector cycle in the combat and prevention of diseases.

## 4 Conclusion

Governments should prioritize health prevention and promotion.^6^ Tackling African and global health challenges offers us the opportunity to improve the well‐being of citizens and initiates a virtuous cycle in which health investments boost economic productivity, thereby providing resources for further health investment.^50^ Through “ubuntu,” a South African concept referring to locally engrained humanity values, we can motivate mutual action toward a collective goal,^3^ which is to make progress across all targets of SDG 3 to the benefit of healthier global citizens.

## Conflict of Interest

The authors declare no conflict of interest.
